# Exodontia Block Course Evaluation: A Review of the Learning Outcomes, Content, and Assessment Practices at a Dental Faculty in South Africa

**DOI:** 10.1111/eje.13130

**Published:** 2025-05-26

**Authors:** Nashreen Behardien, Priscilla Brijlal, Nicolette Vanessa Roman

**Affiliations:** ^1^ Department of Maxillofacial and Oral Surgery Faculty of Dentistry, University of the Western Cape Cape Town South Africa; ^2^ Department of Oral Hygiene, Faculty of Dentistry University of the Western Cape Cape Town South Africa; ^3^ Faculty of Community and Health Sciences University of the Western Cape Cape Town South Africa

**Keywords:** appreciative inquiry, course evaluation, curriculum review, exodontia, tooth extraction

## Abstract

**Introduction:**

Regular curriculum renewal ensures relevant and responsive curricula. Skills development courses, such as for dental extraction procedures, require the same rigorous review, as this skill demands both technical proficiencies and a high level of cognition. In South Africa, the high burden of dental disease, in particular pain and sepsis, relies on competent graduate exodontia (tooth extraction) abilities. However, research on evidence‐based instruction in this field is limited. This action research study emanated from the goal of enhancing the Exodontia Block Course by integrating a teaching and learning strategy proven to develop psychomotor skills. The first step in the broader research study involved evaluating the traditional course. This study thus aimed to evaluate a traditional exodontia block course, gaining insight into its curricular components prior to redesigning it.

**Materials and Methods:**

An interpretive qualitative study employed appreciative inquiry for data collection. A purposive sampling strategy was employed. Six focus group discussions were conducted with homogeneous groups of undergraduate dental students (*n* = 13), clinical teachers (*n* = 10), and dentist practitioners (*n* = 7). Audio recordings were transcribed, and data underwent coding and thematic analysis.

**Results:**

This paper presents the findings pertaining to three curricular elements, namely the objectives, content, and assessment. The larger study identified four main themes: integration of skills and knowledge, block course structure, challenges, and recommendations for improvement. The study found that overall the students were satisfied with the content presented in the course. Recommendations to improve the course, however, were to include the use of elevators as a learning outcome of the course and the inclusion of an additional practical assessment. Challenges associated with the course related to the duration of the course and the presentation of non‐relevant content. The course content revealed discrepancies between the material covered and the intended learning outcomes, with students noting insufficient focus on elevator/luxator use in extractions. Concerns were raised about including non‐exodontia content, including a call for standardised terminology and locally developed teaching materials. Assessment methods were largely well received, but issues emerged regarding unlimited assessment attempts potentially reducing student accountability. Suggestions included limiting assessment opportunities and incorporating OSCE formats for preclinical evaluation to enhance rigour and student learning.

**Conclusions:**

The evaluation highlighted the strengths of the course as well as areas requiring reflection and emendation. Whilst the course was adequate to meet its objectives, areas for redress included instrumentation adaptations such as the combined use of elevators and luxators in intra‐alveolar (simple, or non‐surgical) extractions. A call for standardised terminology, locally developed teaching materials, and a broader array of assessment types was recommended. Additionally, issues emerged regarding unlimited assessment attempts potentially reducing student accountability. The need for future initiatives that focus on developing a nationally standardised competency‐based curriculum was also foregrounded. Follow‐up research would be required to investigate the impact of the implementation of the revised course.

## 1 Introduction

Curriculum review in dental programmes is crucial to ensure best practices for effective skills development, especially in facilitating mastery of invasive procedures such as exodontia. *Exodontia*, a tooth extraction procedure, is defined as ‘the painless removal of a tooth from its investing structures, without causing postoperative prosthetic problems’ [1]. Exodontia necessitates an expedient and judicious approach to ensure patient safety and comfort. Given the elevated litigation risks associated with surgical procedures [2], optimised training and skills development are imperative. With the growing body of literature indicating that physicians and graduate medical education institutions share a collective responsibility, by law, to deliver safe and appropriate care to patients [3], there is increased pressure on academic institutions to develop curricula that ensure optimal training and competency development of students. Empirical evidence within the research context and extending into neighbouring dental schools suggest the need for specialist engagement with the exodontia curricular to align with international practice [4].

Research on exodontia education, although plentiful, lacks comprehensive, robust multi‐stakeholder engagement. The most comprehensive studies are those conducted by Brand et al. [5] and those by MacLuskey and Durham [6]. These studies surveyed dental schools across Europe and the United Kingdom (UK), respectively, regarding their exodontia curricula in their Dentistry programmes. These studies revealed that didactic teaching typically commenced in years 2–3, with considerable variation among curricula. Most schools reportedly relied on textbooks and handouts, while preclinical training predominantly occurred using dental models. Student assessments commonly comprised Objective Structured Clinical Examinations (OSCEs) and workplace‐based assessments [7, 8].

Research aimed at supporting and enhancing exodontia skills curricula globally, and particularly in South Africa, remains exceedingly limited, with few studies offering substantive data on skills development [5, 8, 9, 10, 11]. Thus, evidence‐based standards for teaching and assessment in this domain are notably deficient. The prevailing literature predominantly centres on student perceptions and clinical teaching rather than on the specific elements of the curriculum such as teaching and learning strategies, clinical teaching and training, assessment practices focused on exodontia education, and relevant content.

At the University of Western Cape, a five‐year basic Dentistry degree is offered. During the first 2 years, the programme focuses on the basic sciences, while also providing exposure to fundamental clinical procedures as early as year‐2. In year‐3, however, a significant milestone is reached, with students being introduced to Maxillofacial and Oral Surgery, arguably the most invasive component of the profession. Exodontia skills education begins at the start of the sixth semester, which is midway through year‐3 as per the current curriculum structure. Two learning units within the larger module, Maxillofacial and Oral Surgery I, are designed to cultivate the skills for administering local anaesthetic and exodontia. This takes place through a 4‐day block course, aligning with competencies and programme outcomes. The learning units focus on: evaluating teeth for extraction, administering local anaesthesia, performing exodontia, managing local anaesthesia and exodontia complications, and managing medical emergencies. An excerpt of the module descriptor detailing the curriculum of the Exodontia Block Course (EBC) is depicted in Table 1. The learning outcomes, instructional methods, and assessment strategies are presented.

**TABLE 1:** Excerpt from the module guide: Essential knowledge, skills and values covered in the course.

	Learning outcomes (derived from course objectives)	Teaching and learning strategies employed	Assessment strategies (examples to be considered for future use)
**Knowledge** Demonstrate an understanding of	Instruments used for local anaesthesia Instruments used in exodontia	Didactic lecture—PowerPoint Activity workbook Video clips Demonstrations	Multiple choice qestions (MCQs) Short essay type Oral
	Intra‐oral injection techniques Exodontia principles	Didactic lecture—PowerPoint Video clips Demonstrations	Simulation based assessment OSCE Directly Observed Procedural Skills (DOPS)
	Medical emergencies and Emergency Medicine	Didactic lecture – PowerPoint Simulated patient—Role play	Simulation based assessment
	Complications related to local anaesthesia Complications associated with exodontia	Didactic lecture – PowerPoint Internet information presented in a face‐to‐face lecture	OSCE Short essay type
**Skills** The student must be able to	Perform intra‐oral injections Perform exodontia	Clinical rotation	OSCE Directly Observed Procedural Skills (DOPS)
	Identify and manage complications associated with local anaesthesia and exodontia	Clinical rotation	Simulation based assessment
**Values** The student must be able to	Work effectively in a team or group and take responsibility for his/her decisions and actions; assume responsibility for the decisions and actions of others within well defined contexts; and accept responsibility for the use of resources where appropriate.	Clinical rotation	Work‐based placed assessment Clinical evaluation exercise (mini‐CEX)

The review of the course was initiated by the module coordinator, who is also the principal investigator (PI), given her extensive involvement with the module. The review of the course was the first phase in the larger course redesign, which focused on the integration of deliberate practice principles, an approach that systemises and optimises the teaching and learning of skills [12]. Several factors initiating the review and redesign included the prolonged absence of a thorough, comprehensive review, advancements in educational technology, changes in the profile of dentistry students, a consequence of a merger of two dental schools with significantly different institutional cultures approximately a decade prior, and a recognition of the potential for enhancing clinical skills through the incorporation of scientifically proven strategies for skills development. In the past, the module had operated on an apprenticeship model, but steps were taken to introduce more diverse clinical teaching strategies. The aim of this study was thus to evaluate the EBC offered as part of the Maxillofacial and Oral Surgery module. The objectives were to evaluate the course curriculum by examining its elements, namely the objectives, content, teaching and learning approaches or strategies, assessment, and clinical training—from the viewpoints of key stakeholders, being students, clinical teachers (CTs), and dentist practitioners (DPs). This evaluation sought to identify strengths, weaknesses, and deficiencies in each of the curricular elements and to make recommendations for amendments to the EBC.

## 2 Materials and Methods

### 2.1 Description of the Traditional Block Course

The block course is organised to cover the topics: (i) applied anatomy, (ii) instrumentation and principles of exodontia, (iii) instrumentation and principles of intra‐oral injection techniques, (iv) complications associated with exodontia and intra‐oral injections, (v) drugs commonly used in oral surgery, (vi) prescription writing and (vii) revision of medical emergencies [13]. The course content and outline is presented in Table 2. The course is presented to approximately 12–14 students at a time, using various teaching strategies and instructional methods.

**TABLE 2:** Exodontia block course content and outline.

Exodontia block course outline			
Day 1	Lecture and demo: 08:15–09:45	Day 2	Demo: 08:15–09:00
	Lecture: video clip on injection techniques Lecture: clinical protocol Demo and activity: extraction forceps		Recap: extraction forceps and extraction techniques
	Clinic: 10:00–12:30		Clinic: 09:00–12:30
	Lectures and activity: 13:15–15:15 Lecture: exodontia techniques Lecture and activity: emergency medicine		Lectures: 13:15–15:15 Elevators in dentistry Complications of exodontia Drugs in dentistry and prescription writing
Day 3	Clinic: 08:30–12:30	Day 4	Clinic: 08:30–12:30
			Self‐study: 13:30–15:00
	Video and demo: suturing: 13:15–15:15		Preclinical online assessment 15:00–16:00

Table 2 also illustrates the block course schedule, outlining the sequential delivery of content through didactic teaching (PowerPoint presentation), workbooks, video clips, demonstrations, and clinical practice.

The initial assessment, in the format of a quiz, which took place prior to the contact session, served a dual purpose. It not only evaluates the students' understanding of anatomical content but also encourages them to review previously covered material. Following the contact session, students undergo an assessment, requiring a minimum score of 80% to advance to the clinical training platform. Recognising individual differences in learning pace [14], students are granted multiple attempts to achieve this mark. Additional assessment opportunities are provided within the first week or two following the initial assessment.

### 2.2 Research Design

The study used an interpretive qualitative research design to explore and gain a deeper understanding of the strengths, weaknesses, and deficiencies of the EBC from the perspectives of key stakeholders, including students and CTs. This design is typically underpinned by the constructivist paradigm, investigating and elucidating how participants construct knowledge by interpreting participants' experiences, beliefs, and perspectives [15]. This study was conducted as action research, with the primary investigator (PI), also the module coordinator, aiming to enhance the course by integrating a well‐established teaching and learning strategy, deliberate practice.

#### 2.2.1 Sampling

A purposive sample of undergraduate students, CTs, and DPs was selected for the study. Students who had completed the course were invited to participate. This included three year groups, extending from year‐3 to year‐5. The PI communicated via the class representatives, as well as the learning management system (LMS) informing students of the study and inviting them to participate. Students were requested to contact the PI via email if they were interested in participating. Clinical teachers, who were currently employed in the department, were invited via the CT WhatsApp group chat, while those who were no longer employed in the department but who had been involved in clinical teaching in the EBC were invited via email. Interested parties were requested to contact the PI if they were willing to participate. Dentist practitioners were recruited via a snowballing technique, where the PI reached out to colleagues at the school, at the National Defence Force, and other public health facilities, shared the study information letter via WhatsApp, and asked them to share it with their colleagues. The request was for interested parties to contact the PI indicating their willingness to participate. Once a list of these individuals was compiled, suitable times for the focus group discussion (FGD) were arranged.

The participants were selected based on their different roles and experiences with the EBC and hence the different lenses through which they viewed the EBC. Each of the three groups contributed perspectives on the course through different lenses. These lenses include that of a CT and a course provider, that of a student and client, and that of a DP, who is able to provide a perspective on the value of the course and its translation into the working world. By including students from junior and senior years, this allowed for the inclusion of a broader perspective of the value and benefits gained from the course. Senior students had time to hone the exodontia skills taught in the course. These students had the time to consolidate the learning from the course. Further, a wider range of ages of participants in all groups reduced potential recall bias.

The undergraduate dentistry students (*n* = 13) sample comprised five students in year‐5, four in year‐4, and four in year‐3. These students had completed the EBC during the years 2018, 2019 and 2021, respectively. This sample constituted 4.98% of eligible students. Due to the nature of qualitative research, rich and relevant data was sought from a specific dentistry programme stakeholder group rather than statistical representation [15]. Additionally, the spread of student participants across the various years of study assisted in mitigating the effects of recall bias. Students in year‐3 may recall events of the course more vividly than those in the more senior years. Due to the COVID‐19 pandemic, the year‐3 class concluded their 2020 academic year in March 2021 and not at the end of the 2020 academic year in December 2020. CTs (*n* = 10) who taught in the MFOS department prior to, or at the time of the study, were eligible to participate. Out of the 12 potential participants invited, 10 responded positively. Additionally, a sample of qualified DPs (*n* = 7) who had previously participated in the course, 0–10 years prior, as undergraduate students were eligible for participation. The inclusion of this group was to collect information regarding the value and application of the course in the working world as well as to establish the course's relevance in shaping their professional practice. The DPs comprised general dentists practicing in various private and public sector settings. The sole eligibility criterion was previous participation in the course as an undergraduate restricted to a 10‐year period.

#### 2.2.2 Data Collection Tools, Process and Analysis

Data collection tools included Google Forms and a semi‐structured discussion guide. Demographic information was collected via the Google Form while FGDs served as the primary data collection method. All participants provided consent by completing an online form after they had received written information about the study. Data collection for this evaluative phase of the study was conducted between July 2020 and March 2021. The FGDs explored participants' perspectives on potential course modifications and how these changes could enhance knowledge transfer [16]. The semi‐structured discussion guide was developed based on the principles of Appreciative Inquiry (AI), exploring all the components of the block course as described by Table 3.

**TABLE 3:** A topic guide based on the principles of appreciative inquiry.

The Phases, including a description of appreciative inquiry	Topic guide
	Inquiry into the experiences of the participants regarding the following: Their individual experiences of the various components of the block course An in‐depth inquiry into the specific components of the course ○ Individuals' peak experiences of the course ○ Suggestions for course improvements Questions pertaining to the various teaching strategies Their main wish for an ‘ideal’ block course
**Discovery** (appreciating) What is going well?	Which parts of the block course did you think worked well? Do you think the demonstrations were valuable for those types of clinical activities? How do you feel about the assessments (80% mark to progress to patient treatment; clinical procedures)?
**Dream** (envisioning) What else might be possible?	If you could have one wish regarding the course, what would it be?
**Design** (co‐constructing) What would that look like?	Inquiry into perceptions of teaching and learning strategies, assessments, pace, layout (scaffolding of theory and practical), etc., of the course. The facilitator uses participant responses as a foundation for further probing.
**Destiny/Delivery** (sustaining) What will it take to get there?	How would you like to see the course presented in future?

The question guide was piloted for face validity with a group consisting of two year‐five students and a focus group of CTs. The features of Appreciative Inquiry (AI), as outlined in Table 3, served as the guiding principle for data collection. Table 3 also presents the question guide focused on the components of the course. While AI traditionally stems from organisational development [17], its efficacy in educational research is widely recognised [18, 19, 20]. Notably, two primary models of AI exist: the 4‐D model developed by David Cooperrider [21] and the 4‐I model introduced by Coghlan, Preskill and Catsambas [22]. The two models are similar in principle with both aiming to harness the positive potential within organisations to drive change. The structure of the process however varies slightly between the two with the 4‐I Model combining elements of Design and Destiny into a single phase called ‘Innovate,’ while the 4‐D Model retains these as separate entities. The decision to use the 4‐D Model and not the 4‐I model was based on the use of the 4‐D model in medical education [20, 23, 24] as well as the simplicity of the phases.

**FIGURE 1:**
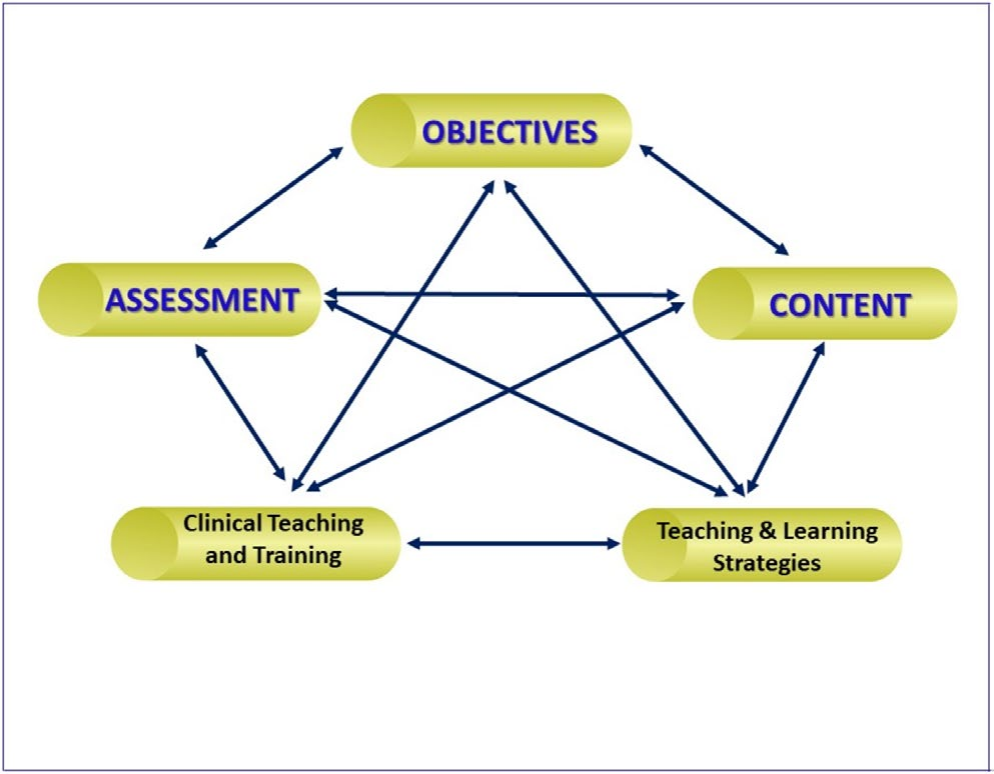
Elements of the curriculum of the course.

The study examined the course's objectives, content, teaching and learning strategies, assessment methods, and clinical training, all of which were infused within the course components (Table 2). Figure 1 illustrates these elements and their interconnections.

A teaching and learning specialist from the university's health sciences faculty facilitated the discussions. This facilitator, well‐versed in AI, was thoroughly briefed on the course before conducting the FGDs. The external facilitator was an experienced qualitative researcher in the field of health professions education who had facilitated and moderated numerous FGDs. The data collection process was validated through a pilot study. Two year‐5 students who participated in piloting the question guide were excluded from the study. The use of an external facilitator was introduced as a means to reduce any potential bias. The FGDs ranged between 50 and 110 min. The duration between the completion of the EBC and the FGD varied among the students at different levels of study. The FGD for the year‐5 group occurred approximately 29 months after having completed the course. Although these students had just graduated and started community service, they participated in the capacity of students. For the year‐3 and year‐4 groups, the duration between completion of the EBC and the FGD was approximately 1 and 22 months, respectively. Given the qualitative nature of the study, the open‐ended questions proved suitable for data generation. The topic guide remained unaltered after the pilot study and was employed flexibly, allowing for exploration of participants' experiences and opinions. Discussions varied in duration because of the focus for each sample as well as the nature of qualitative data collection methods but concluded when no new information emerged or when responses became repetitive, indicating saturation. Focus groups were preferred as a data collection tool over the conventional interview due to their strength in terms of their generative capacity by participants tapping into each other's contributions.

Due to the COVID‐19 pandemic, the study used virtual FGDs, introducing potential biases, such as unreliable internet connections of participants, the inability to use technology adequately, etc. Additional drawbacks included potential unequal engagement, connectivity issues, loss of visual cues due to inactive webcams, and possible exclusion of participants lacking internet access. Online FGDs, however, also offered benefits like reduced costs and wider geographical participation. Despite these limitations, online FGDs proved valuable for continuing research safely during the pandemic. The FGDs of this study were conducted via Zoom and Google Meet platforms. Torrentira [25] advocated for video conferencing to replace in‐person FGDs during COVID‐19. When interpreting the data, it's important to consider the impact of these factors on the findings. Six FGDs were conducted. Each student year group had individual FGDs, whilst the CT sample had two sittings, one group consisting of six participants and the other four. The DP group consisted of seven participants.

The discussions were audio‐recorded and transcribed. All data were transcribed by an independent service provider, with the PI checking it for accuracy. The transcriptions thereafter underwent coding using an open coding method and were analysed following Tesch's eight steps [15]. During the analysis, the PI and external coder familiarised themselves with the data and independently made initial notes about potential patterns and any novel and interesting points raised by the participants. This study used open and inductive coding by systematically working through the entire dataset. The external coder was the primary coder. In the initial phase of open coding, the external coder simplified the data into distinct units of meaning based on the outcomes of the teaching and learning strategies. Each unit was coded, after which some of the codes were condensed. Additionally, the coding was done inductively as the themes were derived directly from the data as no pre‐conceived categories were formed. This was useful in the exploration of new ideas as it related to the teaching and learning strategies. The codes were sorted into potential themes and many were combined to form the overarching themes. These themes were reviewed by the PI, who met on numerous occasions in order to reach consensus on, and finalise the themes. To ensure anonymity, each participant was assigned a code. The coding system used to identify responses or quotations included ‘S’ for the student group, ‘CT’ for the clinical teacher group, and ‘DP’ for the dental practitioner group. The external coder was chosen based on her qualifications as a healthcare professional (social worker and PhD graduate), her experience as an academic at a tertiary education institution in South Africa, and her expertise in supervising qualitative PhDs.

The trustworthiness of the data was ensured through credibility, transferability, and confirmability. *Credibility* was established by the PI's extensive involvement in the data collection and transcription processes. Member‐checking was conducted by the PI by reviewing the data with the participants and ensuring the accuracy of the transcriptions. The topic guide, while allowing for some flexibility, was consistently used to guide the discussion in all of the FGDs. *Transferability* was achieved by examining different aspects of exodontia skill development. The findings of the study have relevance to the development of clinical skills in various medical and allied health professions. *Confirmability* was attained through consensus sessions between the PI and the external coder, focusing on the themes identified during the analysis.

## 3 Results

The evaluation gathered insights from diverse stakeholders including students, educators, and practitioners, providing a comprehensive overview of their collective experiences with the course. Four main themes emerged from this research. A comprehensive table of these results is presented in a previous paper [26]. The study's main findings revealed overall satisfaction among students with the original EBC course (Table 3). Four primary themes emerged: the *integration of skills and knowledge* and *block course structure* showcased the strengths of the course, while the other two themes addressed *challenges and recommendations for improvement*.

### 3.1 Participant Demographics

The student group consisted of five males and eight females, with an age range of 20 to 24 years (mean = 22.7 years). The CT group consisted of four male and six female participants, aged between 27 and 62 years (mean = 36.6 years). Among the CT participants, seven were actively involved in clinical teaching at the time of the study, supplementing their roles as part‐time practising clinicians in the private sector. Notably, CT7, CT9 and CT10 had resigned from their faculty positions at the time of the study, with one having emigrated, one working in the dental public sector, and another pursuing specialisation in Maxillofacial and Oral Surgery. Collectively, the CT group boasted 131 years of clinical teaching experience.

The participants in the DP group were aged between 23 and 33 years (mean = 28 years), with three females and four males. Together, they amassed 34 years of experience. The diverse range of experience within the DP group provided valuable insights into various work environments relevant to the students' training. These practitioners were employed across private and public sectors, the South African National Defence Force, and an academic teaching institution. Table 4 provides additional insight into the characteristics of the CT and DP participants.

**TABLE 4:** Demographic information of the participants.

Clinical teacher (CT) sample (*N* = 10)									
Participant identifier	Characteristics	M	F	Age in years	Number of years qualified	Additional qualification	Hours (p/w) practising as a GD	Hours (p/w) teaching	Years as a CT
		4	6						
CT1	Private practitioner/Academic		X	37	15	PGDip	15	20	10
CT2	Private practitioner	X		35	12	PGDip	30	8	1.5
CT3	Private practitioner		X	36	13	—	40	20	1
CT4	Private practitioner		X	35	12	PGDip	28	12	4.5
CT5	Private practitioner		X	35	13	PGDip	30	16	5.5
CT6	Private practitioner	X		62	30	Oral hygiene; LLB; Hon Oral Surgery	30	20	30
CT7	Private practitioner	X		36	14	MSc(dent)	50	10	7
CT8	Private practitioner	X		27	4	—	48	8	2
CT9	Public sector		X	32	9	PGDip	40	10–12	4
CT10	Specialist in training	X		31	9	MSc(dent)	20	10	4

Dental practitioner (DP) sample (*N* = 7)									
Participant identifier	Characteristics	M	F	Age in years	Years since qualification	Additional qualification			
		4	3	23–33					
DP1	Private practice	x		28	5	PGDip			
DP2	Academic facility		x	26	3	PGDip			
DP3	Private practice; Public dental clinic	x		33	9	—			
DP4	SANDF		x	31	8	—			
DP5	Private practice; Public dental clinic		x	28	4	PGDip Aesthetics			
DP6	Public dental clinic	x		23	1	—			
DP7	Private practice; Public dental clinic	x		27	4	—			

Abbreviations: GD, general dentist; p/w, per week; PGDip, postgraduate diploma in dentistry; SANDF, South African National Defence Force; LLB, law degree; Hon, honours.

### 3.2 Course Curricular Elements

Due to the interconnectedness of curricular components, it is difficult to report on them separately. Hence, this study's findings are structured around objectives and learning outcomes, content, and assessment, with supporting participant quotations included. The results are displayed in a table within the AI framework. Table 5 presents the emerged themes and categories relevant to the learning outcomes, content, and assessment, along with verbatim quotes.

The Discovery Phase outlined the successful features of the course, with the themes *Integration of knowledge and skills* and *Block course structure* showcasing its strengths (Table 3). The Dream Phase yielded the themes *Recommendations for improvement* and *Block course structure*. Lastly, the Design Phase reflected the themes *Challenges associated* and *Recommendations for improvement*, providing foundational insights for course redesign.

### 3.3 Objectives—Translating Into Learning Outcomes

While the broader module goals were evident, the course objectives may have been obscured within. A more explicit focus for the objectives of the course, and how it aligns to the learning outcomes may have been murky. In particular, the detail concerning elevators and luxators as instruments to aid in tooth extraction appeared to be a concern. Further, as described in the content section below, the view of a CT regarding the omission of content related to pharmacology indicates that better defined parameters for the block course should be explored (Table 5).

**TABLE 5:** Table indicating the emerged themes and sub‐themes with the relevant quotes plotted in conjunction with the appropriate curricular elements.

Themes with subthemes	Curriculum elements with quotes supporting the theme		
	Objectives/learning outcomes	Content	Assessment
**Theme: Integration of knowledge and skills**. Sub‐themes: (i) The existing Exodontia Block Course provides a foundation for oral surgery practice; and (ii) The layout (components) of the existing Exodontia Block Course is well planned.	(i) *The Oral Surgery block was well structured. … and is the most practical. Like the lectures aren't like these long things that confuse you. It was simple, straightforward*. (DP6)	(i) *I think the content that was given—it was more than enough because you don't only want to sit in the lab and listen to lectures; we also have to go into clinics. All the lectures that we needed were given before we entered the clinic …. So, everything, everything was covered*. (SV5) (ii) *The timing of the lectures prior to entering the clinics was very important, so that once you do go to the clinic, you have an idea of how to deal with certain situations*. (SV4)	
**Theme: Block course structure**. Sub‐theme: Teaching and learning strategies—Assessment for and of learning			(i) *I think it is, it's a pivotal thing. I mean you can't go into a clinic when you're working with patients, and you've obviously got all these things that could happen if you do the wrong thing or you use the wrong technique. So, you definitely have to know your work*. (SIII4) (ii) *So, I think 80% pushes and drives you to study as well*. (SV1) (iii) *… is to structure the whole assessment completely differently into [a] more OSCE type of setup where students move to different stations rather than looking at a piece of paper. Again, maybe not able to identify something in a 2D form and rather have stations … So perhaps the structure of the assessment, not the content of the assessment, maybe could be improved*. (CT7)
**Theme: Recommendations for improvement**. Sub‐theme: Students' recommendations to improve teaching the skill of tooth extraction: Curriculum issues—Use of elevators	(i) *I think luxators definitely needs to be touched on a bit more in fourth year. They can maybe just be shown a bit in third year to understand because just seeing the picture…* (SV7) (ii) *… and often, like a root fracture, does a patient come in with like half a crown, and then we will need to use elevators, some cryers or something to get it out. And, it's like the first time you're holding a cryer*. (SIV2)		
**Theme: Recommendations for improvement**. Sub‐theme: Students' recommendations to improve teaching the skill of tooth extraction: Curriculum issues—standardising the content and terminology		(i) *So, one thing that I feel students would appreciate is if all supervisors are on one page when it comes to what students were taught*. (SIV4)	
**Theme: Challenges**. Sub‐theme: Reliance on teachers—Ownership of learning			(i) *Students take chances. They know they're gonna have to repeat that assessment until they pass. So, for them, it's a joke at the end of the day. They have that attitude of “I don't care, I'm here; my supervisor won't fail me. I have multiple chances to pass.”* (CT9)
**Theme: Challenges** Sub‐theme: Block course duration	(i) *I think it's important to obviously highlight what the goals of the block … you know, at the start of the block because my perception of it is to prepare the student to be able to manage oral surgical procedures clinically and so, over bombarding them with information that might not be appropriate to their clinical skills* (CT7)	(i) *I think the pace of it is a bit too much personally, I think that because we bring a lot of content into it, we might be rushing through elements that need a bit more time for the student. And so, if we take away unnecessary [content], we might be able to slow the pace for a better understanding of basic concepts*. (CT7)	
**Theme: Recommendations for improvement**. Sub‐theme: Students' recommendations to improve teaching the skill of tooth extraction: Curriculum issues—Use of elevators		(i) *There was just one thing that I would have liked. That's just personal preference is the use of elevators. So, in our block, we just use the normal forceps and then at a later stage, we get to learn how to use the elevators*. (SIV4) (ii) *We weren't exposed to elevators as much. And as a result, we're struggling with elevators this year*. (SIV3)	
**Theme: Recommendations for improvement**. Sub‐theme: Clinical teachers' recommendations to improve the skill of tooth extraction. Curriculum issues—additional practical assessment.			(i) *So I think a more practical assessment, whatever form it is*. (CT7) (ii) *… we need to relook at certain things in terms of making sure that we teach students earlier on; so, in third year, focus on teaching rather than on assessment, which will come in your fourth [or] in your fifth year*. (CT7)

The course, however, seemed to achieve its objectives or learning outcomes, as evidenced by the theme: *Integration of knowledge and skills*. Several factors appeared to contribute to this success, a sentiment echoed by both the student and DP groups. The simplicity of the course structure appeared to be a significant contributing factor to its success: ‘It was simple, straightforward (DP6)’. *Additionally, success in terms of meeting the course outcomes, were expressed by: I think the block was really beneficial. Before the block, I would never would have been able to do any of the stuff* (SIV2).

The inclusion of the DP group lends credence to the course, as the outcomes of the course are applied in practical, real‐world contexts by this group:

[T]he most valuable things that dental school that could have taught me was taught in that block course. … I am rural [in a rural setting]; there's nobody close to help me. So, when something happens, I have to …. [I]t taught me to think on my feet and also to keep calm. (DP4)

… it prepares you to be able to do safe extractions outside whether it be in a clinic or private setting. (DP1)

The use of the elevator/luxator in the exodontia procedure emerged as a notable concern among the students. They suggested incorporating the use of this instrument as an outcome, necessitating adjustments to the course content of the EBC. *I feel like we weren't exposed to elevators as much. And as a result, we're struggling with elevators this year*. (SIV3).

I think it was a good block. But maybe adding small things like, for example, either elevator demonstration would improve it a little bit more. (SV4)

### 3.4 Content

A discrepancy between the course content and the intended learning outcomes was observed as suggested in the ‘objectives’ section. This was noted as an omission of detailed teaching of the use of elevators/luxators in intra‐alveolar extractions. Year‐4 and 5 students echoed this sentiment. Understandably, year‐3 students had not had adequate exposure to the clinical teaching and training platform at the time of the FGD and thus did not offer any perspectives on the issue.

I feel like if, if the students are taught, like they are taught about forceps from the get go, they should be taught about elevators and luxators from the get go, so that by the time they get to final year, it's a normal you just perfect on your work round up. (SIV4)

The CT group expressed a concern regarding the inclusion of content not directly linked to exodontia skills development. The example of the lecture on ‘Drugs in dentistry and prescription writing’ was quoted as an example. The sense was that the time used for this lecture could be better spent acquiring and developing the exodontia skill. Only content pertaining to exodontia skills and its complications should be presented in the course.

The student group expressed a desire for standardisation of terminology within the course. In the Dream Phase, recommendations were made for standardising the terminology. The student sample indicated that it was best practice for all CTs to use the same terminology during the block course as this was at a foundational level, and although CTs came from different backgrounds and experiences, the standardisation of terms would reduce confusion and support their learning. The CT group emphasised the importance of developing local content and avoiding the use of American‐developed teaching material, as the terminology often differed. In addition to the varied terminology, the injection techniques presented in the American‐developed videos differed marginally from the techniques taught in the course. These sentiments were expressed by SIV4 and CT7:

But one thing that I … feel students would appreciate is if all supervisors, in that discipline, are on one page when it comes to what students were taught [standardisation of terminology]. (SIV4)

…the content was good but maybe not appropriate to [the] South African setting. If I can, if I can think back to the lecture on injection technique, uhm, [it] was really good, but the videos were, well, Americanised. So, they were demonstrating techniques that were maybe not necessary to what we were doing at the time at the faculty. (CT7)

### 3.5 Assessment

Overall, the assessments that took place within the EBC were well received. However, a concern was raised regarding some students' lack of responsibility for their learning.

They [students] know they're gonna have to repeat that assessment until they pass. So, for them, it's a joke at the end of the day. …. I have multiple chances to pass. (CT9)

Both the CT and student groups proposed setting a limit on the number of opportunities for assessment. Given that assessment drives learning [27], having a finite number of chances may encourage students to prepare more diligently. This idea is corroborated by SV1's remark, indicating that the 80% minimum requirement serves as an incentive to study:

So, I think 80% pushes and drives you to study as well. You don't want to fail in front of your colleagues or your classmates as well.

**FIGURE 2:**
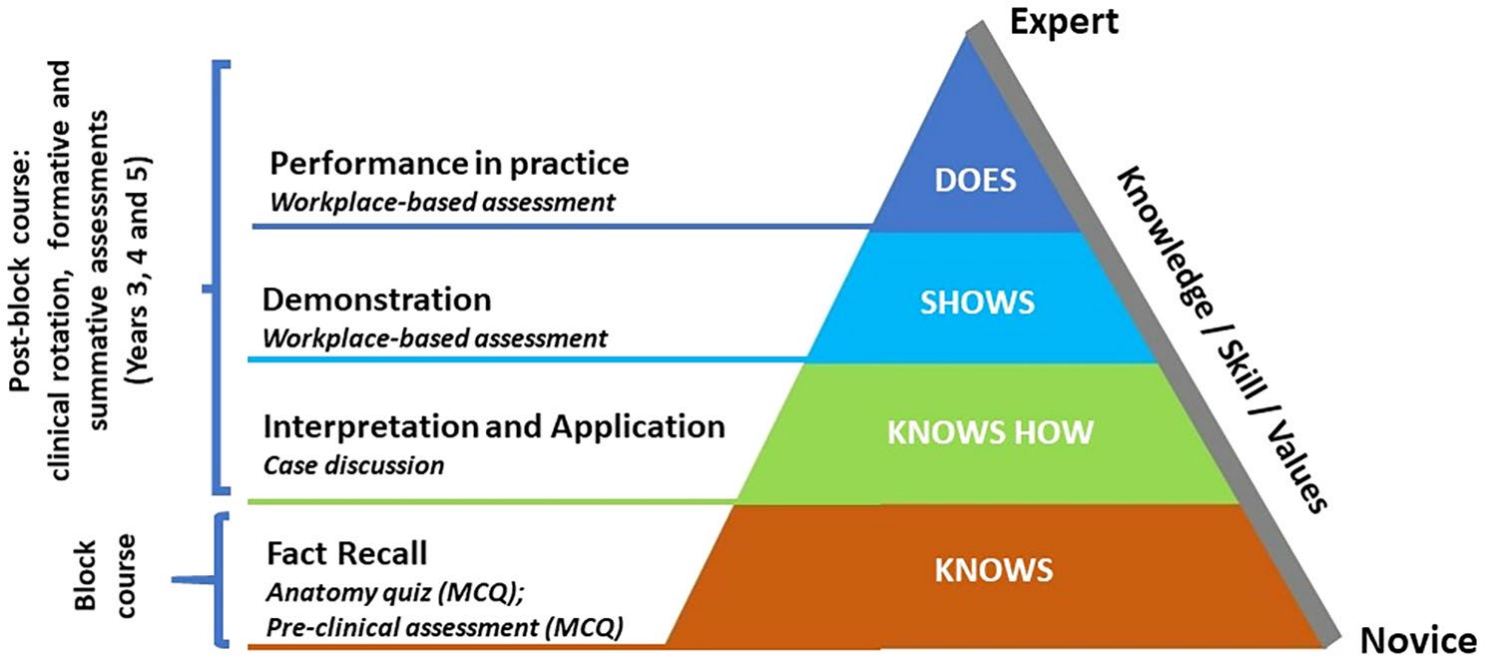
Miller's Pyramid illustrating skills assessment strategies used during and after the Block Course (adapted from Miller's Pyramid of Clinical Competence) [28].

Figure 2 illustrates the levels of knowledge and skills acquisition using Miller's Pyramid, along with examples of assessment strategies linked to the EBC. It showcases the developmental approach to learning, utilising assessment as a guiding tool for learning.

Since the acquisition of a skill reflects a continuum of learning, it is understandable that senior students reflected on their EBC experience and the subsequent years' Oral Surgery clinical rotation as a single entity. The assessments of the EBC primarily reflected the competencies expected in the first level of Miller's Pyramid (Figure 1). CT7's perspective, advocating for the incorporation of an OSCE format for preclinical assessment, is seen as a means to enhance assessment rigour and consequently, bolster student learning.

The sub‐theme *Teaching and Learning Strategies—Assessment for and of learning* elicited conflicting views on clinical assessment and feedback. Some students deemed feedback solely on clinical performance sufficient, suggesting no allocation of marks for performance. Additionally, a disconnect between student expectations or self‐assessment and the marks assigned by CTs led to students feeling demoralised, as noted by SIII1: ‘She was so disheartened about the mark that she had gotten, which in hindsight, is not really what it comes down to’. Nonetheless, there was a consensus between CTs and students that the focus for junior students on the clinical training floor should emphasise more teaching and less assessment. The following quotation reflects this perspective:

… in a sense where you get some sort of feedback to say, “Okay, this is where you went wrong. I would have given you this mark if you had done so and so.” Uhm, but block week, I don't think it would be good to get marked. (SIII1)

Assessments that allow deep and strategic learning approaches as opposed to surface learning would be beneficial in the clinical teaching and training context.

## 4 Discussion

Learning serves as the cornerstone of education. The evaluation of the EBC curriculum aimed to enhance instructional components that influence skill acquisition, drawing on theories such as cognitive constructivism and experiential learning. The utilisation of the AI framework effectively facilitated discussions, generating abundant rich data. Although elevator extraction methods were theoretically introduced, practical application was lacking. Senior students, particularly those in years 4 and 5, have advocated for more comprehensive instruction in this area. Multiple studies have corroborated students' desire for enhanced teaching on elevator techniques, which are crucial for patient safety, clinician comfort, and procedural versatility [5, 29]. The addition of a second instrument for intra‐alveolar tooth extraction in the course merits further exploration. It's important to note that the technical skills required for forceps differ significantly from those needed for elevators and luxators. A scaffolded approach to teaching these three techniques may be beneficial, with elevator skills building upon the foundation of forceps proficiency. Elevators and luxators can serve as valuable adjuncts to forceps in tooth extraction. By using an elevator or luxator to loosen a tooth from its socket, clinicians can significantly reduce the force required to separate the tooth from its investing structures. Elevators are used to engage the tooth, using the adjacent alveolar bone as a fulcrum, and lifting the tooth, thus tearing the periodontal ligament. Luxators, however, are used in a gentler nature, slowly rocking the tooth and using a cutting surface to cut the periodontal ligament. In this study, students routinely appeared to use the term ‘elevators’ as a collective term to include luxators. It is possible that they view the two instruments, of which the basic anatomy (handle, shank, working surface) is almost identical, as the same instrument. This would have to be explored since the principle of use of a luxator, differ to that of an elevator. This may and do not tend to distinguish the ‘luxator’ as a separate instrument with a slightly different anatomy and use. With the course being only 4 days long, an extension of the course, as also suggested by participant samples, may have to be beneficial to including elevator and luxator education.

The standardisation of terminology significantly enhances information retention, especially for students from diverse language backgrounds. Consistent use of terms reinforces learning through repetition. When students encounter the same vocabulary across lectures, clinical sessions, and interactions with CTs, it strengthens their recall and deepens their understanding of the content. Standardised terminology also minimises the risk of misinterpretation. When all lecturers and CTs use consistent language, key information is conveyed more clearly and accurately. This is particularly crucial in technical fields like dentistry, where precision is paramount. Moreover, consistent terminology reduces cognitive overload for students. Instead of expending mental energy deciphering varying terms for the same concepts, learners can focus more on understanding the underlying ideas. This streamlined approach to language not only facilitates better comprehension but also creates a more efficient learning environment overall [30, 31].

Careful selection of assessments should consider student level, competencies being evaluated, design transparency, assessor calibration, and patient safety. Assessment of clinical skill should ideally be formative in nature when a new skill is being developed. When considering the nature of skills development formative assessment may benefit the development of the skill. In the broader context of the research, the deliberate practice model emphasises the role of repeated practice with feedback. A hybrid assessment format encompassing gaming activities, low‐risk simulation assessment, and clinical assessment, tailored to student level, could be beneficial. Ensuring assessor calibration is essential for maintaining the quality, validity, and fairness of assessments [32].

Addressing the lack of student accountability could involve embedding the university's graduate attribute of ‘scholarship’ – a critical attitude towards ‘knowledge’ – into the hidden curriculum of the broader dentistry programme. The hidden curriculum in health professions plays a pivotal role in cultivating the expected behaviour and knowledge from students. Physical spaces like tea rooms, activities such as journal club meetings, and modelling professionalism by academic and clinical staff are implicit methods of nurturing graduate competencies.

Furthermore, reviewing teaching and learning activities to increase opportunities for self‐directed learning (SDL) within the broader module could be valuable. SDL has the potential to foster autonomy and independence. This behaviour may typically be seen in students who are self‐motivated, able to manage their time optimally, and seize all opportunities to improve their skill set such as voluntary outreach projects [33]. Offering a range of opportunities such as voluntary after‐hour outreach programmes, access to skills laboratories for clinical practice, and assignments that develop independent learning skills, accountability, responsibility, and assertiveness could be beneficial. These could be facilitated through visits to private dental practices and public health facilities.

Recommendations for improving the assessment component of the EBC were diverse, including suggestions for adopting an OSCE format, for example. The CT group advocated for more rigorous face‐to‐face assessments, emphasising a definitive shift from the online format. While the online format offered convenience, a major drawback was the reduced rigour in assessing clinical procedural skills. Face‐to‐face assessments, such as OSCEs, allowed for a more comprehensive demonstration of learning, enabling deeper student engagement and assessing competence in micro skills development. Assessments aligned with Bloom's taxonomy can effectively measure affective, psychomotor, and cognitive domains, providing novices with comprehensive evaluations that enhance learning, engagement, skill acquisition, and safe clinical preparation. Looking ahead, fostering a partnership between dental schools and specialist surgery groups can further enrich the curriculum by incorporating contemporary practices [4].

The concept of reflexivity of the PI in this study is a consideration since the PI is also the module co‐ordinator. To this end, the PI has included numerous strategies, as presented in the methodology, to ensure the validity and integrity of the study.

## 5 Conclusion

This study provided a comprehensive understanding of the intricacies and interplay of and between the elements of the EBC curriculum. Overall, students responded positively to the course content and assessments, although CTs identified some assessment practices in need of review. Notably, students specifically recommended equal emphasis on elevator extraction techniques alongside forceps. A concerted effort is to be made to incorporate the content and teaching and learning strategies to adequately teach the principle of use and skills for the use of elevators/luxators in intra‐alveolar extractions.

The involvement of student participants allowed for the inclusion of student perspectives as end users, granting them agency in the educational process. While patient feedback is invaluable in medical education, a limitation of this study was the exclusion of patients who underwent exodontia as a sample group. Nevertheless, the findings of this study are poised to advance the knowledge base in exodontia teaching, since baseline information is required before improvements can be made. The findings from this study augment the current body of knowledge in terms of the type of course delivery used for teaching exodontia, as well as the views of exodontia education from the perspectives of various stakeholders. In the context of this research, the baseline information will provide a basis for incorporating the new teaching and learning strategy, deliberate practice and potentially benefiting procedural skills education in other sectors.

## Author Contributions

N.B. contributed 60%, and P.B. and N.V.R. contributed 40% jointly.

## Ethics Statement

Ethics approval for this study, which was part of a PhD study, was obtained from the Biomedical Research Ethics Committee (BM19\10\23) of the university. The study conformed to the ethics guidelines of the Declaration of Helsinki. All participants provided informed consent prior to participating in the study.

## Conflicts of Interest

The authors declare no conflicts of interest.

## Data Availability

Data is shared in this article; additional data information can be requested from the author.
